# Coupling between Blood Pressure and Subarachnoid Space Width Oscillations during Slow Breathing

**DOI:** 10.3390/e23010113

**Published:** 2021-01-15

**Authors:** Agnieszka Gruszecka, Magdalena K. Nuckowska, Monika Waskow, Jacek Kot, Pawel J. Winklewski, Wojciech Guminski, Andrzej F. Frydrychowski, Jerzy Wtorek, Adam Bujnowski, Piotr Lass, Tomislav Stankovski, Marcin Gruszecki

**Affiliations:** 1 Department of Radiology Informatics and Statistics, Medical University of Gdansk, 80-210 Gdansk, Poland; agruszecka@gumed.edu.pl; 2 Department of Human Physiology, Medical University of Gdansk, 80-210 Gdansk, Poland; magda.nuckowska@gmail.com (M.K.N.); pawel.winklewski@gumed.edu.pl (P.J.W.); 3 Institut of Health Sciences, Pomeranian University of Slupsk, 76-200 Slupsk, Poland; monika.waskow@apsl.edu.pl; 4 National Centre for Hyperbaric Medicine, Institute of Maritime and Tropical Medicine, Medical University of Gdansk, 81-347 Gdynia, Poland; jkot@gumed.edu.pl; 5 Department of Computer Communications, Faculty of Electronics, Telecommunications and Informatics, Gdansk University of Technology, 80-233 Gdansk, Poland; wojciech.guminski@pg.edu.pl; 6 NIRTI SA, 53-676 Wroclaw, Poland; andrzejfrydrychowski@wp.pl; 7 Department of Biomedical Engineering, Faculty of Electronics, Telecommunications and Informatics, Gdansk University of Technology, 80-233 Gdansk, Poland; jerzy.wtorek@eti.pg.edu.pl (J.W.); bujnows@biomed.eti.pg.gda.pl (A.B.); 8 Department of Nuclear Medicine, Medical University of Gdansk, 80-210 Gdansk, Poland; piotr.lass@gumed.edu.pl; 9 Department of Molecular Spectroscopy, Faculty of Mathematics, Physics and Informatics, University of Gdansk, 80-309 Gdansk, Poland; 10 Faculty of Medicine, Ss. Cyril and Methodius University in Skopje, 1000 Skopje, North Macedonia; t.stankovski@ukim.edu.mk; 11 Department of Physics, Lancaster University, Lancaster LA1 4YW, UK

**Keywords:** time series, nonlinear dynamics, coupling

## Abstract

The precise mechanisms connecting the cardiovascular system and the cerebrospinal fluid (CSF) are not well understood in detail. This paper investigates the couplings between the cardiac and respiratory components, as extracted from blood pressure (BP) signals and oscillations of the subarachnoid space width (SAS), collected during slow ventilation and ventilation against inspiration resistance. The experiment was performed on a group of 20 healthy volunteers (12 females and 8 males; BMI =22.1±3.2 kg/m${}^{2}$; age 25.3±7.9 years). We analysed the recorded signals with a wavelet transform. For the first time, a method based on dynamical Bayesian inference was used to detect the effective phase connectivity and the underlying coupling functions between the SAS and BP signals. There are several new findings. Slow breathing with or without resistance increases the strength of the coupling between the respiratory and cardiac components of both measured signals. We also observed increases in the strength of the coupling between the respiratory component of the BP and the cardiac component of the SAS and vice versa. Slow breathing synchronises the SAS oscillations, between the brain hemispheres. It also diminishes the similarity of the coupling between all analysed pairs of oscillators, while inspiratory resistance partially reverses this phenomenon. BP–SAS and SAS–BP interactions may reflect changes in the overall biomechanical characteristics of the brain.

## 1. Introduction

Variations in beat-to-beat interval evoked by rhythmic breathing that occurs during a respiratory cycle were described in the middle of the nineteenth century [1]. During spontaneous breathing, the heart rate (HR) decelerates during expiration and accelerates during inspiration [2]. It is a well known phenomena in physiology—respiratory sinus arrhythmia (RSA). When the breathing frequency slows down to 6 breaths/min, the amplitude of the heart rate variability (HRV) reaches its maximum. Consequently, a breathing frequency of 0.1 Hz is often referred to as a ‘resonance frequency’ [3,4].

Since ancient times, slow breathing has been believed to have a beneficial effects on mental relaxation through the enhancement of autonomic, cerebral and psychological flexibility (reviewed in [5]). Nevertheless, the available scientific data of good quality is scarce. For instance, Hinterberge et al. (2019) demonstrated that decelerated breathing at 0.1 Hz promotes synchronisation between breathing, RSA and slow cortical potentials. Respiratory activity crucially regulates the flow of cerebrospinal fluid (CSF, see the review in [6,7]).

Gruszecki et al. [6] recently proposed that a CSF pulsatility flow could be represented by subarachnoid space (SAS) width oscillations. It is possible to measure the SAS width oscillations in humans with a non-invasive NIR-T/BSS method. The measurement of the changes of the SAS width is possible because the NIR-T/BSS method uses an infrared radiation which propagate in the SAS filled with translucent CSF [8,9,10,11]. The SAS space could be treated as an optical duct for infrared radiation [8,9]. To validate the method, Frydrychowski et al. [12] compared measurements of magnetic resonance imaging against NIR-T/BSS. They compared changes of the SAS width between two volunteers’ positions: supine vs. abdominal-lying. Based on regression analysis, a high correlation between these two methods was shown (R=0.81, *p* < 0.001).

We recently demonstrated that ventilation at 0.1 Hz diminishes cardiac component of the blood pressure (BP) and SAS oscillations. For cardiac frequency interval, the SAS and BP oscillators are less coherent during slow breathing. An enhanced SAS and BP amplitude noticed during ventilation at 0.1 Hz is generated by respiration; this effect is further augmented by application of inspiratory resistance. The SAS and BP oscillators are more coherent at respiratory frequency when breathing at 0.1 Hz. We used a wavelet analysis with the Morlet mother wavelet for the delineation of the cardiac and respiratory components of the SAS and BP signals and a further assessment of the signals’ amplitudes [13].

Consequently, in the current study, we aimed at assessing phase coupling and cardiac frequency accelerations of the SAS and BP signals during slow breathing and slow breathing with inspiratory resistance. The present study is a follow up analysis of the data collected by Nuckowska et al. [13]. To tackle this problem, we analysed the phase dynamics of interacting oscillations. This allowed us to study the coupling functions[14,15]. Coupling functions describe how an interactions occur and are manifested. They describe in detail the mechanism behind the causal interactions between the systems. Various methods were designed to restore a coupling functions from obtained data [16,17,18,19,20,21]. These have enabled applications in different scientific fields, including chemistry [22], climate [23], secure communications [24], mechanics [25] and social sciences [26]. Coupling functions have proved to be especially suitable for studying oscillatory interactions in physiology for cardiorespiratory and cardiovascular interactions [16,27,28,29] and in neuroscience for various aspects of connectivity [30,31,32,33,34]. In the present paper, coupling functions is applied for the first time to the cardio–respiratory interactions from the SAS and BP observational signals.

We hypothesised that slow breathing may strengthen the respiratory–cardiac coupling of the BP and CSF. In particular, we expected that slow breathing may: (1) increase the strength of the respiratory–cardiac coupling in SAS and BP; (2) augment the strength of the coupling between the respiratory component of the BP mode and the cardiac component of the SAS; (3) enhance the strength of the coupling between the respiratory component of the SAS and cardiac component of the BP; and (4) decrease the similarity of the coupling between the respiratory and cardiac components of the SAS and BP, between the respiratory component of the BP and cardiac component of the SAS and between the respiratory component of the SAS and the cardiac component of the BP. We also speculated that respiratory resistance applied during slow breathing may partially restore the above mentioned similarity.

## 2. Results

**Figure 1:**
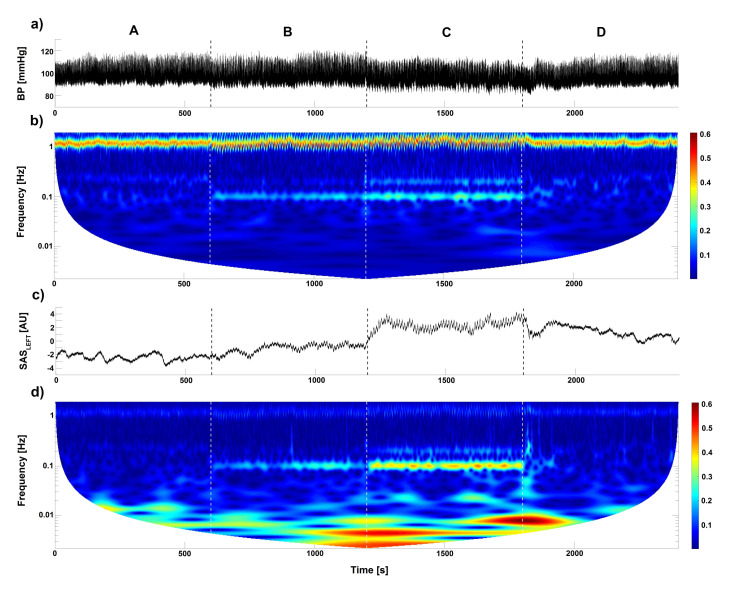
Time evolution of (**a**) BP and (**c**) ${\mathrm{SAS}}_{\mathrm{LEFT}}$ width (left hemisphere) signals. The four letters (A–D) correspond to stages of experimental protocol: (A) baseline, ventilation with normal respiration rate; (B) 6 breaths/min ventilation; (C) 6 breaths/min ventilation with resistance; and (D) recovery, ventilation with normal respiration rate. (**b**,**d**) The amplitude of wavelet transforms for (**b**) BP and (**d**) ${\mathrm{SAS}}_{\mathrm{LEFT}}$ width signal estimated for whole recording (40 min). The signals were measured for one subject.

With the analysis of the wavelet transform and coupling functions, we investigated different impact on cardiovascular processes between slow and resistance breathing. To learn about it, the experimental protocol consisted of four different stages: (A) baseline, ventilation with normal respiration rate; (B) 6 breaths/min ventilation; (C) 6 breaths/min ventilation with resistance; and (D) recovery, ventilation with normal respiration rate. Figure 1a,c shows BP and ${\mathrm{SAS}}_{\mathrm{LEFT}}$ signals collected from one of the volunteers. In turn, Figure 1b,d illustrates the amplitude of wavelet transform estimated for the BP and ${\mathrm{SAS}}_{\mathrm{LEFT}}$ signals. Figure 1d (Figure 1b) shows the ${\mathrm{SAS}}_{\mathrm{LEFT}}$ (BP) signal. For both signals, we can see a similar cardiac component (about 1 Hz). Furthermore, a respiration components of 0.1 Hz (6 breaths per minute) are visible. These components appear during both stages (B and C) of experimental procedure.

In Figure 1, we can see that the respiratory component during slow breathing cause an increases of oscillations of the SAS and BP. The wavelet amplitude for both signals is the highest when inspiratory resistance was introduced. All of these indicate a strong influence from respiration to the cardiac rhythms. The estimated coupling functions help to measure this interaction.

**Figure 2:**
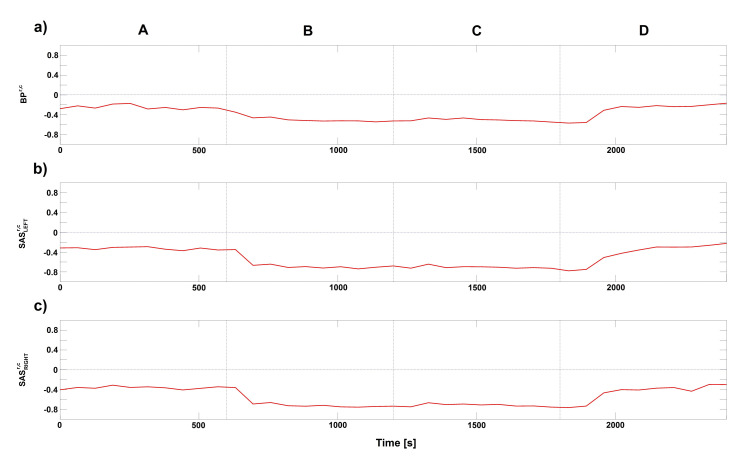
Time evolution of group-averaged a directionality index D(t) estimated for the phases of the respiratory and cardiac signals components for BP, SAS${}_{\mathrm{LEFT}}$ and SAS${}_{\mathrm{RIGHT}}$. To estimate D(t), Equation (5) was used. The four letters (A–D) correspond to stages of experimental protocol: (A) baseline, ventilation with normal respiration rate; (B) 6 breaths/min ventilation; (C) 6 breaths/min ventilation with resistance; and (D) recovery, ventilation with normal respiration rate. Negative value of D(t) means that respiration component of signal drives the cardiac component of signal for whole period of experiment.

After extraction and separation of the components and phases ($\phi_{Card}$ and $\phi_{Resp}$) from the collected signals, the cardio–respiratory interactions were studied through their coupling functions (CFs). First, we estimated directional index D(t). The obtained results were plotted on the Figure 2 for the BP, ${\mathrm{SAS}}_{\mathrm{LEFT}}$ and ${\mathrm{SAS}}_{\mathrm{RIGHT}}$ signals. The D(t) were estimated for all four stages of the procedure. For all three signals, the value of D(t) is negative. Additionally, the value of D(t) decreases during second and third stages of procedure for all three signals. Negative value of D(t) means that respiration mode drives the cardiac mode and this is a predominant direction of coupling for those signals. This is evidence of strong influence from respiration to the cardiac rhythms. In our analysis, we considered only this direction of coupling.

**Figure 3:**
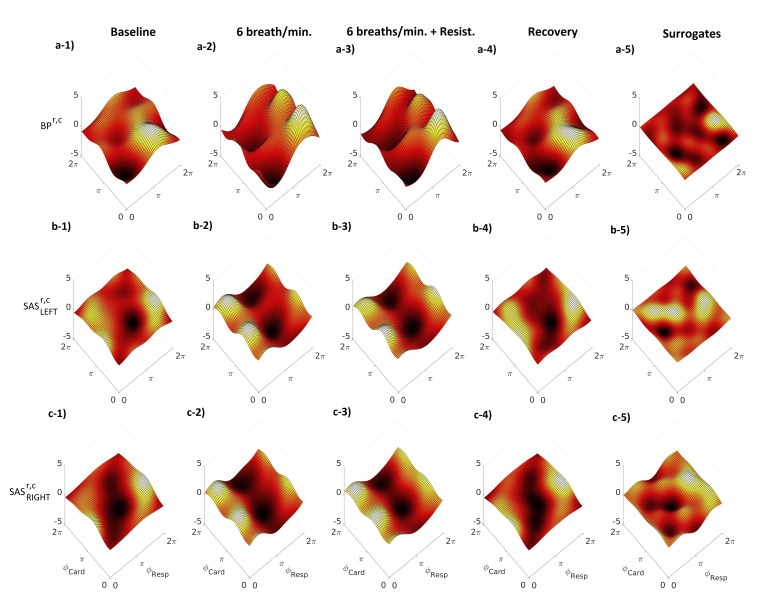
Group-averaged coupling functions between the phases of respiratory $\phi_{Resp}$ and cardiac $\phi_{Card}$ oscillations estimated for the same signals. Columns correspond to different stages of the experiment: baseline, 6 breaths/min, 6 breaths/min, + resistance and recovery. Additionally, to find insignificant values of coupling functions, a surrogate threshold was estimated. Rows show the coupling functions $q^{r,c}$ between the phases of the respiratory and cardiac signals for BP, SAS${}_{\mathrm{LEFT}}$ and SAS${}_{\mathrm{RIGHT}}$, respectively. Note that, when coupling functions lose sinusoidal shape along $\phi_{Resp}$ axis (baseline and recovery stages), weaker interaction between respiration and cardiac components of considered signal is observed.

Figure 3 illustrates the averaged cardio–respiratory CFs for the BP, ${\mathrm{SAS}}_{\mathrm{LEFT}}$ and ${\mathrm{SAS}}_{\mathrm{RIGHT}}$ signals. The CFs were estimated for all four stages of the procedure. Additionally, we estimated for all three signals the cycle phase permutation surrogates (panels a–5, b–5 and c–5). It is clearly visible that the amplitude of the coupling for the surrogate is smaller and insignificant when we compare it to the results for the stages of the procedure. The first row of Figure 3 (from a–1 to a–4) shows that the form of the CF for the BP signal, for all stages of procedure, resembles a sinusoidal shape along the respiratory phase axis $\phi_{Resp}$. Along the cardiac phase axis $\phi_{Card}$, the CF has nearly constant shape. This means that the mechanism of a direct coupling, where the influence of $\phi_{Resp}$ is the predominant one. The sinusoidal shape is more enhanced during slow breathing with or without resistance. A similar shape can be observed for the SAS signals (both hemispheres), but the sine-like shape of the CF is shifted along the $\phi_{Resp}$ axis (cf. Figure 3 from a–1 to a–4, from b–1 to b–4 and from c–1 to c–4).

**Figure 4:**
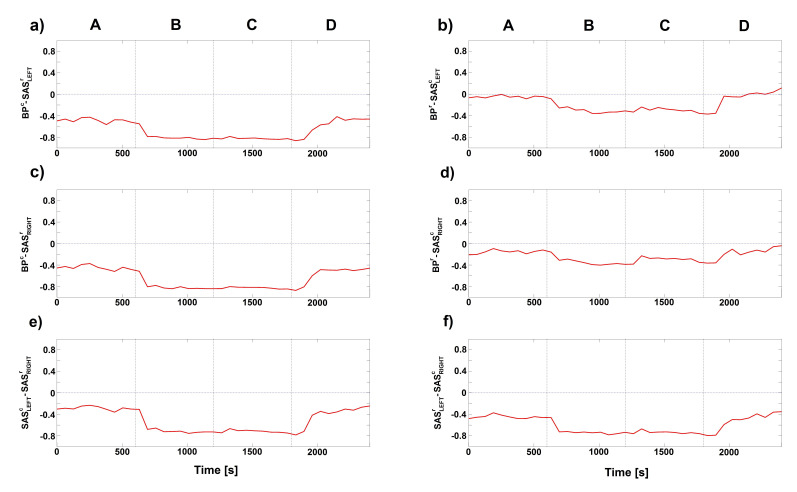
Time evolution of group-averaged a directionality index D(t) (see Equation (5)) estimated for the phases of the respiratory and cardiac signals for different combination of measured signals. The four letters (A–D) correspond to stages of experimental protocol: (A) baseline, ventilation with normal respiration rate; (B) 6 breaths/min ventilation; (C) 6 breaths/min ventilation with resistance; and (D) recovery, ventilation with normal respiration rate. Negative value of D(t) means that respiration component drives the cardiac mode of considered signal for whole period of experiment.

**Figure 5:**
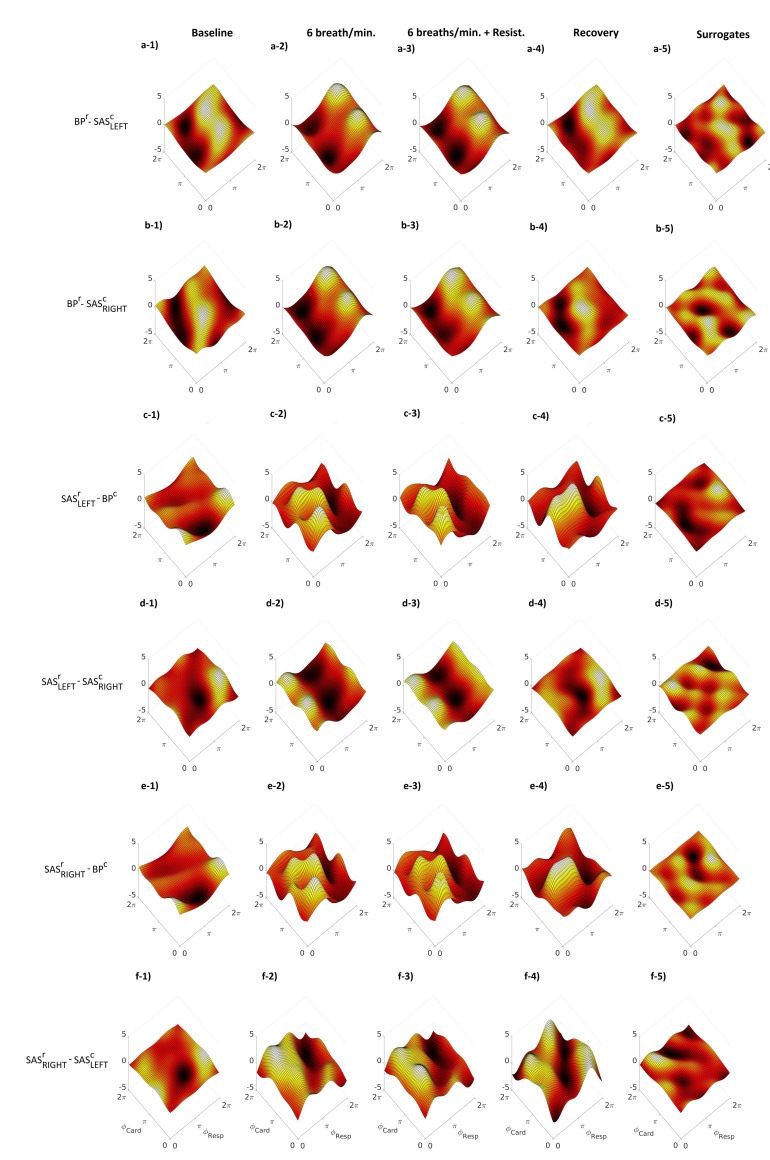
Group-averaged coupling functions between the phases of respiratory $\phi_{Resp}$ and cardiac $\phi_{Card}$ oscillations estimated for different combination of components of measured signals. Columns correspond to: baseline, 6 breaths/min, 6 breaths/min + resistance and recovery. Additionally, to find insignificant values of coupling functions, a surrogate threshold was estimated. Rows show the coupling functions $q^{r,c}$ between the respiratory and the cardiac components for different combinations of measured signals (BP-SAS, SAS-BP and SAS-SAS). Note that a sinusoidal shape of coupling functions along $\phi_{Resp}$ axis is connected with stronger interaction between respiration and cardiac oscillations of considered signals.

Similar conclusions can be found for the D(t) and CFs estimated for cardio–respiratory interactions for different signals, e.g., ${\mathrm{BP}}^{r}-{\mathrm{SAS}}_{\mathrm{LEFT}}^{c}$, ${\mathrm{BP}}^{r}-{\mathrm{SAS}}_{\mathrm{RIGHT}}^{c}$, as shown in Figure 4 and Figure 5. Negative value of D(t) (Figure 4) indicate that respiration mode drives the cardiac mode and this is a predominant direction of coupling for those signals. The general conclusion for CFs is that the ${\mathrm{BP}}^{r}-{\mathrm{SAS}}_{\mathrm{LEFT}\mathrm{or}\mathrm{RIGHT}}^{c}$ (Figure 5a,b) estimated functions have the well known direct sine-shape form, while the other direction ${\mathrm{SAS}}_{\mathrm{LEFT}\mathrm{or}\mathrm{RIGHT}}^{r}-{\mathrm{BP}}^{c}$ (Figure 5c,e) is much less pronounced, with lower and greatly varying functional forms. The third direction between the two sides LEFT-RIGHT of SAS (Figure 5d,f) is again relatively well pronounced, although the form of their coupling function seems shifted from that of ${\mathrm{BP}}^{r}-{\mathrm{SAS}}_{\mathrm{LEFT}\mathrm{or}\mathrm{RIGHT}}^{c}$.

**Figure 6:**
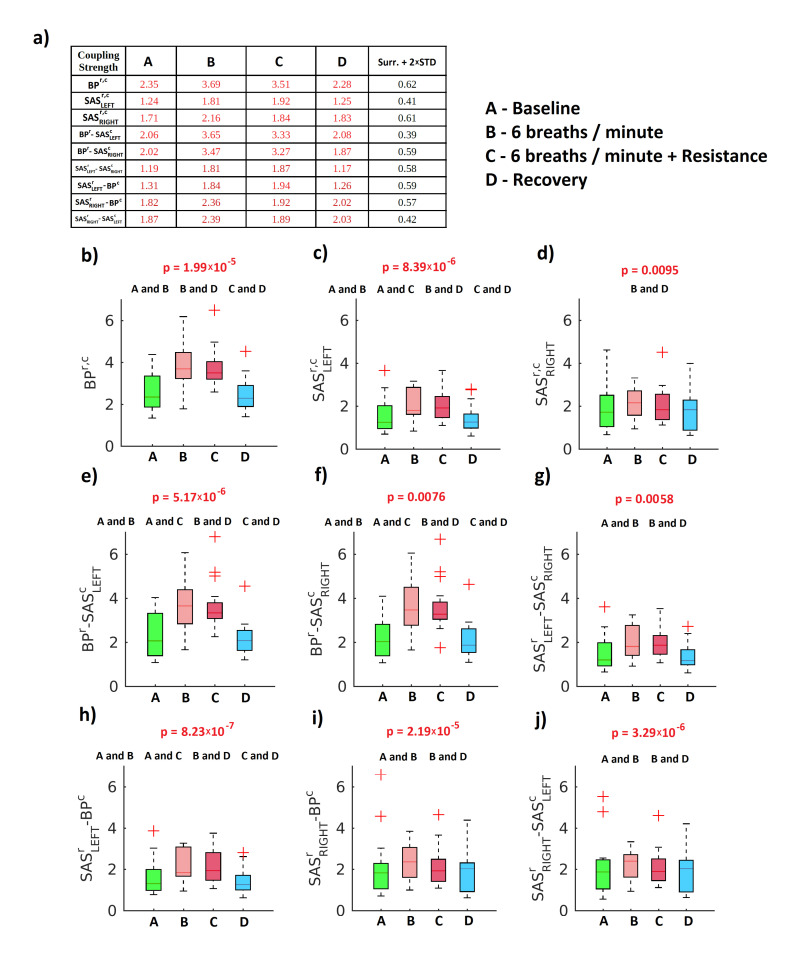
Statistics for coupling strength. (**a**) Median values for the coupling strength σ for four stages of protocol ((A) baseline, ventilation with normal respiration rate; (B) 6 breaths/min ventilation; (C) 6 breaths/min ventilation with resistance; and (D) recovery, ventilation with normal respiration rate) and the coupling surrogate threshold. In red (black) median values significantly (not significantly) different from mean surrogate plus two standard deviations. (**b**–**j**) Box-plots illustrating the distribution within each stage of the protocol of the strength σ of the coupling between the respiratory and cardiac components. The Friedman test was used to estimate the *p* value. ‘A and B’ means that Stage A (baseline) differs significantly from Stage B (6 breaths/min), and similarly for ‘B and D’, etc. Red crosses correspond to outliers.

Figure 6 illustrates the medians of the coupling strength σ. All median values of σ for all stages of experimental procedure are higher than the surrogate threshold (mean plus two standard deviations (Table a of Figure 6)). To find differences between the values of the coupling strength, for all stages of procedure, we used the Friedman test. The results of a box-plot analysis are displayed in panels from b to j. We found that all obtained results were statistically significant (p<0.05). From the obtained results we can conclude that the coupling strength grows during slow and resistance breathing for all considered combinations of respiration and cardiac components extracted from the registered signals. This means that the RSA is more enhanced during slow and resistance breathing due to the fact that the inhale and exhale last longer than during spontaneous breathing during baseline or recovery. Additionally, with resistance, breathing requires greater effort from the volunteers. This was partially observed also in cardio–respiratory CF in linear ramped contentiously varying breathing [20,35] as well as in sine and aperiodic varying breathing [29], where the CF were higher for lower respiration frequency.

**Figure 7:**
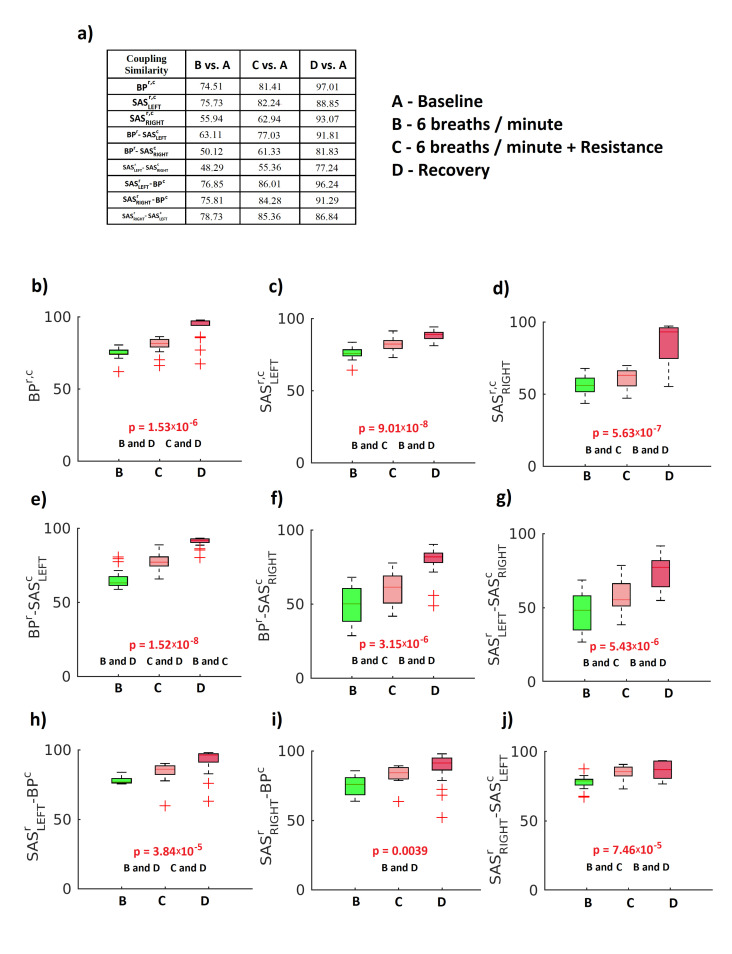
Statistics for coupling similarity. (**a**) Median values for the coupling similarity for three stages of protocol (B–D) compared to baseline stage (A) ((A) baseline, ventilation with normal respiration rate; (B) 6 breaths/min ventilation; (C) 6 breaths/min ventilation with resistance; and (D) recovery, ventilation with normal respiration rate). (**b**–**j**) Box-plots illustrating the distribution of the coupling similarity within each stage of protocol for respiratory and cardiac components of the measured signals. The Friedman test was used to estimate the *p* value. ‘B and D’ and similar symbols are the same as in the previous figure. Red crosses correspond to outliers.

There was also estimated the coupling similarity index ϱ (Figure 7) for similarity of form of the coupling functions between the baseline and other stages of the experimental procedure (6 breaths/min ventilation, 6 breaths/min ventilation with resistance and recovery). Panel (a) of (Figure 7) illustrates the table and all other panels show median values of ϱ. The results of the Friedman test are displayed in panels b to j of (Figure 7). For all cases, the results were statistically significant (p<0.05). The values of ϱ show that the shape of the CFs during the recovery stage are the most similar to the baseline stage. This means that the cardiovascular system comes back to its initial state after slow breathing, both with or without resistance.

## 3. Discussion

There are several new findings of the present study. In particular, slow breathing: (1) increases the strength of the respiratory–cardiac coupling in the SAS and BP; (2) augments the strength of the coupling between the respiratory component of the BP mode and the cardiac component of the SAS; (3) enhances the strength of the coupling between the respiratory component of the SAS and cardiac component of the BP; and (4) decreases the similarity of the coupling between the respiratory and cardiac components of the BP and SAS, between the respiratory component of the BP and cardiac component of the SAS and between the respiratory component of the SAS and the cardiac component of the BP. Respiratory resistance applied during slow breathing does not affect the coupling strength of the assessed functions, however it partially restores their similarity.

**Figure 8:**
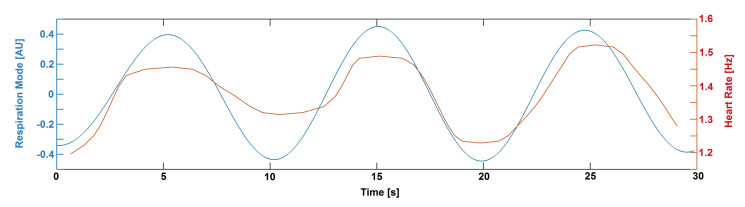
Time evolution (30 s segment) of respiratory component (**blue line**) and heart rate (**red line**) extracted from the BP signal from one of the volunteers.

The first of our findings (the increase of the strength of the respiratory–cardiac coupling in the SAS and BP) relates to the well-known phenomenon of RSA. The slower function (respiratory component) affects the quicker oscillator (HR; [15]). As the coupling strength increases, HR (during inspiration) acceleration is observed, and, when the coupling decreases (during exhaust), HR decelerates (Figure 3 and Figure 8). The coupling between the respiratory and cardiac components was previously described by the authors of [27,28]. However, Iatsenko et al. [27] analysed the ECG signal while Ticcinelli [28] registered the laser Doppler flowmetry signal representing microvascular flow. Consequently, either of the coupling functions between oscillations generated directly by the heart (ECG) or propagated to the smallest vessel (laser Doppler flowmetry) were assessed. In this study, we analysed for the first time BP and SAS oscillators and demonstrated that both are affected by slow ventilation and slow ventilation with resistance.

Transfer of augmented arterial pulsatility to the brain tissue and the CSF may have an impact on the brain structure and function [7,36,37]. It is believed that arterial stiffness associated with ageing and hypertension augments this pulsatility and increases the risk of cerebrovascular events (reviewed recently in [38]). Other authors however postulate that decreased pulsatility of cerebral vessels may deteriorate the CSF flow through perivascular spaces in the brain, which is crucial for the clearance of metabolic waste, including β-amyloid [39,40]. Consequently, from a homeostatic perspective, it is tempting to speculate that ‘brain pulsatility’ is tightly controlled to maintain just ‘the right amount of pulsatility’, while the underlying mechanisms remain largely obscure.

To investigate this further, here we also used an analysis based on coupling functions, which describe in detail how the cardiac oscillations are affected due to a causal influence from the respiration oscillations. In particular, the mechanism described by the form of the coupling functions explains how, with higher values of the coupling function, the cardiac oscillations are accelerated, while during lower values of the coupling function, the cardiac oscillations are decelerated due to the influence of the respiration. Our results in Figure 3 and Figure 5 demonstrate that the cardio–respiratory coupling functions a direct sine-wave shape, closely associated with the RSA mechanism.

The sine-like shape can be observed also for SAS signals (both hemispheres), but the CF is shifted along the $\phi_{Resp}$ axis. This effect is clearly visible in both Figure 3 and Figure 5. We are not able to determine whether the observed differences are related to physiological phenomena (e.g., brain autoregulatory buffering) or different locations (e.g., different propagation times from the rhythm generators: heart and lung) of the registration devices. BP was registered from the finger while the SAS signal was collected from the head. In future studies, we plan to collect transcranial doppler signals to investigate further.

Another interesting feature is the fact that the coupling functions for the SAS signals for both hemispheres have similar shapes. In the previous study [6], it was found that the correlation between the SAS oscillations in the right and left hemisphere varied significantly between subjects. Additionally, differences in phase coherence in almost all considered frequency intervals were identified. Those differences were recorded at resting state. During this experiment, the volunteers were focused on a task, namely to breathe as instructed by the computer screen, and we did not observe any differences in the coupling functions between the two hemispheres. Consequently, focus on breathing augments CSF pulsatility synchrony between brain hemispheres.

In Figure 5, we can also see that the shape of the CF for ${\mathrm{BP}}^{r}-{\mathrm{SAS}}^{c}$ is different from that of ${\mathrm{SAS}}^{r}-{\mathrm{BP}}^{c}$. The shape of the CF for ${\mathrm{BP}}^{r}-{\mathrm{SAS}}^{c}$ has a direct sine-wave shape, closely associated with the RSA mechanism, while the CF for ${\mathrm{SAS}}^{r}-{\mathrm{BP}}^{c}$ does not have a sine-wave shape. A very similar effect can be observed for the CFs for the SAS signals. The shape of the CF for ${\mathrm{SAS}}_{\mathrm{LEFT}}^{r}-{\mathrm{SAS}}_{\mathrm{RIGHT}}^{c}$ has a direct, shifted sine-wave shape, closely associated with the RSA mechanism, while the CF for ${\mathrm{SAS}}_{\mathrm{RIGHT}}^{r}-{\mathrm{SAS}}_{\mathrm{LEFT}}^{c}$ does not have a sine-wave shape. Forced inspiration is associated with venous blood movement from the head toward the thorax, while CSF is drawn away from the thorax into the brain. The reverse happens during expiration [41]. CSF flows and brain tissue properties are increasingly recognised as an important component of the brain’s homeostatic control [42,43]. Thus, BP−SAS and SAS−BP interactions may reflect changes in the overall status of the biomechanical characteristics of the brain. Due to the fact that the brain is placed in a skull, even small changes in CSF flows may affect the properties of the brain tissue and BP/CSF interdependencies. ${\mathrm{SAS}}_{\mathrm{RIGHT}}-{\mathrm{SAS}}_{\mathrm{LEFT}}$ interactions, in turn, potentially underly ongoing hemisphere–specific mental processes and related changes in brain perfusion [44].

We recently demonstrated that the BP respiratory oscillations during slow breathing diminish the amplitudes of the SAS cardiac pulsatility [13]. The second finding of the present study (slow breathing increases the strength of the coupling between the respiratory component of the BP and the cardiac component of the SAS ) implies that the cardiac SAS pulsatility accelerations and decelerations are more pronounced during the respiratory cycle. The CSF oscillations of respiratory origin potentially act as a low-pressure counter pulsating system that moves CSF and may help to clean accumulations of debris in the peri-venous spaces [45]. Inspiration augments and exhalation diminishes the venous outflow from the head [46]. Thus, enhanced inspirations during slow breathing and quicker HR accelerations may further support CSF flow, while diminished cardiac oscillations amplitude at the same time may have protecting effect on the vessels.

Evidence is accumulating that intracranial pressure oscillations are associated with sympathetic activity in a reciprocal way to arterial baroreflex. Consequently, even a small increase in intracranial pressures augments sympathetic firing, with a subsequent BP elevation, which in turn promotes cerebral perfusion pressure stability over time [47,48]. Therefore, it is not surprising that the SAS respiratory oscillation effects the BP cardiac accelerations and decelerations (the third finding of our study: slow breathing enhances the strength of the coupling between the respiratory component of the SAS and the cardiac component of the BP). More pronounced CSF movements resulting from bigger pressure gradients generated by respiratory movements (these mechanisms are reviewed in detail in [7,13]) lead to enhanced changes in sympathetic activity and corresponding changes in HR.

The similarity of the coupling between all pairs of oscillators is diminished during slow breathing. By implementation of inspiratory resistance, this effect was partially reversed. Slow breathing is associated with augmented lung volume, which results in Hering–Breuer reflex stimulation. The role of Hering–Breuer reflex, mediated by activation of stretch receptors, is lung protection from overinflation. All of these stimulate vagus afferents [49]. However, the additional effort to inhale, during slow breathing with resistance, increases sympathetic activity [50]. Our previous studies show consequently that the BP-SAS relationship is stabilised by the sympathetic nervous system [7]. In particular, BP-SAS amplitude coherence at cardiac frequency was stabilised. Changes in autonomic nervous system activity less affected phase coherence [13,51].

The results of our study are consistent and extend previous reports. All of these indicates the credibility of the applied findings and techniques. For instance, when subjects breath with inspiratory resistance, we observed HR accelerations [52] and increase in blood oxygenation [53] or during slow breathing a decline in the BP [54,55]. Moreover, the volunteers managed not to hyperventilate and avoid subsequent decrease in ${\mathrm{EtCO}}_{2}$. These allowed for precise delineation of the regulatory mechanisms.

Beneficial effects of slow ventilation are increasingly recognised by western medicine [56], particularly as the nonpharmacological enhancement of therapy in patients suffering from diabetes mellitus type II, hypertension and heart failure [55,57,58]. Our study describes several previously unknown mechanisms related to interactions among lungs, BP and CSF. Future longitudinal studies in selected patients groups with extensive use of neuroimaging methods should verify whether the reported mechanisms can be translated into medical procedures. In particular, the effect of CSF motion on perivascular spaces [42,59] and overall brain biomechanics [43] should be investigated.

Using coupling functions, we unveiled several mechanisms describing bidirectional dynamic interactions between the blood pressure (BP) and oscillations of SAS during normal breathing, slow ventilation and slow ventilation with resistance. BP-SAS and SAS-BP interactions may reflect changes in the overall biomechanical characteristics of the brain. Since CSF flows and properties of the brain tissues are becoming the vanguard at the novel approach to brain research, our study increases the understanding of the underlying processes. Moreover, slow breathing synchronises the SAS oscillations between brain hemispheres. In addition, slow breathing diminishes the similarity of the coupling between all analysed pairs of oscillators, but inspiratory resistance partially reverses this phenomenon.

## 4. Materials and Methods

### 4.1. Subjects

The experimental protocol was similar to in our previous study [13]. Briefly, the experiment was performed on a group of 20 healthy volunteers (12 females and 8 males; BMI =22.1±3.2 kg/m${}^{2}$; age 25.3±7.9 years) The experimental protocol and the study were accepted by the Ethics Committee of the Medical University of Gdansk, Poland (NKBBN/265/2016), and the experiment was carried out in accordance with the Helsinki recommendations for the ethical treatment of human subjects. Before the experiment, all subjects were instructed about the study’s aims, and all volunteers gave written informed consent to take a part in the experiment. We asked the participants to avoid from consuming cocoa, coffee, tea and any meals or drinks containing methylxanthine for at least 8 h before the experiment. Additionally, for at least 24 h before the experiment, the participants were asked to avoid an alcohol. Before the beginning of the experiment, all volunteers rested for 10 min in a quiet and comfortable room.

### 4.2. Experimental Design

The experiment was performed in a quiet and warm (18–20 °C) room [13]. Before the experiment, all participants learnt how to breathe with the use of the study equipment [13]. By avoiding very deep ventilation, the volunteers kept away from hyperventilation. After this practise, the participants were resting. Next, the baseline stage of experiment were begun. The experimental procedure consisted of ventilation with normal respiration rate and 6 breaths/min ventilation followed by 6 breaths/min ventilation with resistance [13]. Each part of procedure lasted 10 min. The rate of breathing was dictated by visual marks displayed on a screen. A modified, single-use Threshold IMT device (Phillips-Respironics, Best, The Netherlands) controlled the increased inspiratory resistance [13]. The inspiratory resistance from 2 to 40 cm $H_{2}O$ can be set up by the device consisting of a mouthpiece and a container. The demanded pressure of inspiratory resistance was equal 20 cm $H_{2}O$ [13].

### 4.3. Measurements

The finger-pulse photoplethysmography (CNAP, CNSystems Medizintechnik AG, Graz, Austria) was used for continuous measurements of HR and BP. The brachial arterial pressure was used to calibrate BP from the finger. SAS Monitor (NIRTI SA, Wroclaw, Poland) was used to register changes of SAS width from both hemispheres. Recently, Gruszecki et al. [6] provided a detailed description of the SAS Monitor. A medical monitoring system (Datex-Ohmeda, GE Healthcare, Wauwatosa, WI, USA) was used to measure SaO${}_{2}$ (oxyhaemoglobin saturation) and EtCO${}_{2}$ (end-tidal CO${}_{2}$).

All signals were simultaneously registered for 40 min. To import the collected signals, powerlab (AD Instruments, Colorado Springs, CO, USA) and LabChart Pro were used. Before the analysis, the collected signals were detrended by the moving average technique and normalised (subtracting their mean and dividing by their standard deviation).

### 4.4. Mathematical Analysis

To learn about the interactions in the cardiovascular system, we conducted the following analyses. At first, we found the existence and estimated the strength of the oscillation using the wavelet transform. Then, we decomposed and extracted the oscillations. To do this, a bandpass Butterworth filter was used. To find the interaction between the extracted components, we calculated the coupling functions through the use of dynamical Bayesian inference [20,60]. All these methods are explained below.

#### 4.4.1. Wavelet Analysis

The wavelet transform (WT) is a time–frequency analysis method. This is a perfect mathematical tool suitable for analysing biological signals. Additionally, the WT uses the logarithmic scale for the frequency, thus low frequencies have higher resolutions. The wavelet transform is given by equation:

(1) $$\begin{array}{c} W\left(s,t\right)=\frac{1}{\sqrt{s}}\int_{0}^{\infty }\phi \left(\frac{u-t}{s}\right)g\left(u\right)du, \end{array}$$

where g(u) is the time series, W(s,t) is the wavelet coefficient and ϕ is the Morlet mother wavelet, scaled by the factor *s* and translated in time by *t*. The Morlet mother wavelet is defined by:

(2) $$\begin{array}{c} \phi \left(u\right)=\frac{1}{\sqrt[{4}]{\pi }}exp\left(-i2\pi u\right)exp\left(-0.5u^{2}\right), \end{array}$$

where $i=\sqrt{-1}$. The Gaussian shape of the Morlet wavelet guarantee a good localisation of events in frequency and time [61]. During this study, we were interested in analysing only the frequency interval (0.07–2 Hz) because we wanted to focus only on cardiac and respiratory activity [62]. The wavelet coefficients W(s,t) are complex numbers, $X\left(\omega_{k},t_{n}\right)=X_{k,n}=a_{k,n}+ib_{k,n}$. This allows the amplitude ($|X_{k,n}|\;=\sqrt{a_{k,n}^{2}+b_{k,n}^{2}}$) and the phase $\left(\theta_{k,n}=\arctan\left(\frac{b_{k,n}}{a_{k,n}}\right)\right)$ to be considered separately.

#### 4.4.2. Decomposition and Preprocessing

A standard bandpass Butterworth filter was applied to extract and separate the cardiac and respiration components from the collected signals. The Butterworth filter is a type of signal processing filter designed to have a frequency response as flat as possible in the passband [63]. We considered only two frequency intervals which correspond to respiration and cardiac activity. Stefanovska et al. [62] showed that intervals 0.145–0.6 and 0.6–2 Hz correspond to the respiration and cardiac functions, respectively. The respiration interval was extended to 0.1 Hz due to the fact that during two stages of experimental procedure the breathing rate was equal 6 times per minute. This rate corresponds to 0.1 Hz. In the reconstruction of the coupling function, we used the phase domain; therefore, we first extracted the phase time-series of the two signals. We did this by first calculating the Hilbert transform of the signals to get the protophase, and then we applied a protophase-to-phase transformation [25].

#### 4.4.3. Modelling of Coupling Functions

The purpose of the applied analysis was to model the data with coupled phase oscillators [64]. To extract the coupling functions of interest, we used dynamical Bayesian inference (DBI). The method helps follow the time-varying behaviour [20,65,66]. To learn about influence of each oscillator on the others, we decomposed the system into a group of phase oscillators which interact. The method helps estimate the behaviour of the system, i.e., the effective coupling function [16,24].

To learn about interactions between oscillators, many methods can be applied [67,68,69,70,71,72]. To find the underlying mechanisms responsible for these interactions, the dynamical Bayesian inference (DBI) [20,60,73] can be used.

To tackle the inverse problem, of determining the coupling connections from a measured signal, the system was modelled as a pair of coupled phase oscillators [64,74]. This system can be defined by two differential stochastic equations:

(3) $$\begin{array}{c} \dot{\phi_{i}}\left(t\right)=\omega_{i}\left(t\right)+q_{i}\left(\phi_{i},\phi_{j},t\right)+\xi_{i}\left(t\right) \end{array}$$

with *i* = 1 and *j* = 2. A coupling function $q_{i}$ of the oscillators’ phase dynamics and its natural frequency $\omega_{i}$ can be used to determine the instantaneous frequency ${\dot{\phi }}_{i}$ of each oscillator. This represent the coupling configuration. The Gaussian white noise $\xi_{i}$ is the stochastic part of Equation (3), while the deterministic periodic part of this equation can be expressed as [75,76]:

(4) $$\begin{array}{c} \dot{\phi_{i}}\left(t\right)=\sum_{k=-K}^{K}c_{k}^{\left(i\right)}\Phi_{k}\left(\phi_{1},\phi_{2}\right). \end{array}$$

This part is characterised by the time-varying $c_{k}^{\left(i\right)}$ parameters. In our studies, the order of the Fourier expansion was set to *K* = 2. Exploiting Bayes’ theorem, the mathematical procedure calculates for each time window the most likely set of parameters *c* by separating the noise and propagating the information carried by the previous time-window [20,60]. From the inferred parameters *c* and their appropriate basis functions $\Phi_{k}\left(\phi_{1},\phi_{2}\right)$ (i.e., a set of sine and cosine Fourier functions), we could extract the separate coupling functions of interest $q_{i}\left(\phi_{i},\phi_{j},t\right)$. A detailed description with practical tutorial and software of dynamical Bayesian inference can be found in [77].

A 2π×2π phase grid can be used to represent a coupling function which express an interaction between a pair of oscillators [20,76,78]. Three measures calculated to simplify quantitative comparisons obtained results: (i) the *directionality index* D(t) (5); (ii) the *coupling strength* σ (6); and (iii) the *polar similarity*ρ (7).

*Directionality index:* The directionality index D(t) is defined as [20]:

(5) $$D\left(t\right)=\left(c_{2}-c_{1}\right)/\left(c_{1}+c_{2}\right).$$

For simplicity and clarity, we limited the above equation to two oscillators. If D∈[−1,0) (D∈(0,1]), the second (first) drives the first (second).

*Coupling strength:* The coupling strength $\sigma_{i,j}$ from oscillator *i* to oscillator *j* is:

(6) $$\sigma_{i,j}=\sqrt{\sum_{k=-K}^{K}(c_{k}^{\left(i,j\right)}{)}^{2}}.$$

All parameters are defined below Equation (4). This parameter inform about the amount of influence that the phase of oscillator *i* exerts on the frequency of oscillator *j*.

*Polar similarity:* A similarity can be quantified by estimation of the correlation between the coupling functions $q_{1}$ and $q_{2}$[16]. The similarity modulus $|\rho_{q}|$ can be defined as:

(7) $$|\rho_{q}|=\frac{\langle {\tilde{q}}_{1}\;{\tilde{q}}_{2}\rangle }{|{\tilde{q}}_{1}\left|\;\right|{\tilde{q}}_{2}|}\times 100,$$

where the tilde ~ is the deviation from the mean and the angular brackets indicate averaging over the 2π×2π grid. The range of |ρ| is from 0% to 100%. In this way, the similarity index is a direct measure of the form of the coupling functions, i.e., the mechanism of the underlying interaction, and as such it is a unique measure of the coupling function.

#### 4.4.4. Statistical Analysis and Surrogates

To estimate the differences between four stages of the experimental procedure, we used a non-parametric Friedman test. Next, if p<0.05, we used the Tukey test to find the differences between four stages of the experimental procedure.

Additionally, the cycle phase permutation surrogates were used to validate the results for the coupling functions. The cycle phase permutation surrogates are phase surrogates. Detailed description of this surrogate can be found in [79].

## Data Availability

The data presented in this study are available on request from the corresponding author. The data are not publicly available due to privacy.
